# Distinct MicroRNAs Expression Profile in Primary Biliary Cirrhosis and Evaluation of miR 505-3p and miR197-3p as Novel Biomarkers

**DOI:** 10.1371/journal.pone.0066086

**Published:** 2013-06-12

**Authors:** Masashi Ninomiya, Yasuteru Kondo, Ryo Funayama, Takeshi Nagashima, Takayuki Kogure, Eiji Kakazu, Osamu Kimura, Yoshiyuki Ueno, Keiko Nakayama, Tooru Shimosegawa

**Affiliations:** 1 Division of Gastroenterology, Tohoku University Hospital, Sendai, Japan; 2 Division of Cell Proliferation, Tohoku University School of Medicine, Sendai, Japan; 3 Department of Gastroenterology, Yamagata University Faculty of Medicine, Yamagata, Japan; Emory University School of Medicine, United States of America

## Abstract

**Background and Aims:**

MicroRNAs are small endogenous RNA molecules with specific expression patterns that can serve as biomarkers for numerous diseases. However, little is known about the expression profile of serum miRNAs in PBC.

**Methods:**

First, we employed Illumina deep sequencing for the initial screening to indicate the read numbers of miRNA expression in 10 PBC, 5 CH-C, 5 CH-B patients and 5 healthy controls. Comparing the differentially expressed miRNAs in the 4 groups, analysis of variance was performed on the number of sequence reads to evaluate the statistical significance. Hierarchical clustering was performed using an R platform and we have found candidates for specific miRNAs in the PBC patients. Second, a quantitative reverse transcription PCR validation study was conducted in 10 samples in each group. The expression levels of the selected miRNAs were presented as fold-changes (2^−ΔΔCt^). Finally, computer analysis was conducted to predict target genes and biological functions with MiRror 2.0 and DAVID v6.7.

**Results:**

We obtained about 12 million 32-mer short RNA reads on average per sample and the mapping rates to miRBase were 16.60% and 81.66% to hg19. In the statistical significance testing, the expression levels of 81 miRNAs were found to be differentially expressed in the 4 groups. The heat map and hierarchical clustering demonstrated that the miRNA profiles from PBC clustered with those of CH-B, CH-C and healthy controls. Additionally, the circulating levels of hsa-miR-505-3p, 197-3p, and 500a-3p were significantly decreased in PBC compared with healthy controls and the expression levels of hsa-miR-505-3p, 139-5p and 197-3p were significantly reduced compared with the viral hepatitis group.

**Conclusions:**

Our results indicate that sera from patients with PBC have a unique miRNA expression profile and that the down-regulated expression of hsa-miR-505-3p and miR-197-3p can serve as clinical biomarkers of PBC.

## Introduction

MicroRNAs (miRNAs) are small endogenous RNA molecules of 19 to 24 nucleotides that control the translation and transcription of targeting mRNAs by base-pairing to the complementary sites [1] [2] [3] [4]. The expression of miRNAs in serum is reported to be stable, reproducible and consistent among individuals of the same species [5]. So far, specific expression patterns of serum miRNAs were identified as a fingerprint for numerous diseases and cancers [6] [5]. The serum miR-122 levels are elevated in patients with liver damage due to chronic hepatitis B (CH-B) and C infection (CH-C) [7] [8]. In addition, the miR-122 and miR-34a levels are positively correlated with the disease severity in CH-C and non-alcoholic fatty-liver disease [9]. However, there are some reports that miR-122 expression in healthy controls was significantly higher than that in patients with hepatitis C virus (HCV) infection [10]. Li et. al. described that 13 miRNAs were differentially expressed in hepatitis B virus (HBV) serum and that miR-25, miR-375 and let-7f could be used as biomarkers to separate a HBV-positive hepatocellular carcinoma (HCC) group from HBV-negative HCC [11]. However, little is known about the expression profile of miRNAs in autoimmune disease such as primary biliary cirrhosis (PBC).

PBC is female predominant, progressive autoimmune disease characterized by immune-mediated destruction of the intrahepatic bile ducts. The serological marker of PBC is the presence of anti-mitochondrial antibody (AMA) directed against the E2 subunit of the pyruvate dehydrogenase enzyme complexes located in the inner mitochondrial membrane [12] [13] [14]. The etiology of PBC is considered to be a combination of genetic predisposition and environmental triggers [15]. Particularly, concerning genetic predisposition, previous studies reported that common genetic variants at the HLA class II, IL12A, IL12RB2, STAT4, IRF5-TNPO3 and IKZF3 had significant associations [16] [17] [18] [19] [20] [21]. Recently, genome-wide association study in Japanese population showed TNFSF15 and POU2AF1 as susceptibility loci [22]. Several GWAS data suggested the important contributions of several immune pathways to the development of PBC. However, the results have differed among the study groups [21]. The diagnosis of PBC is established based on the following criteria: (1) biochemical evidence of cholestasis; (2) the presence of AMA; and (3) histopathologic evidence of nonsuppurative cholangitis and destruction of the interlobular bile ducts [23]. Though diagnostic criteria have been determined, the eventual progression to biochemically and clinically apparent disease is unpredictable. Many patients are recognized at an earlier stage of disease and respond well to medical therapy, while some patients will require liver transplantation [24] [25]. To revolutionize the diagnosis, treatment and prognosis of PBC, new biomarkers seem to be feasible, and miRNAs are emerging as highly tissue-specific biomarkers with potential clinical applicability [26] [6].

In this study, we employed a strategy of using Illumina small-RNA sequencing for the initial screening followed by quantitative reverse transcription PCR (qRT-PCR) validation to analyze serum samples, which were arranged in multiple trial and testing sets. Additionally, computer analysis was conducted to predict target genes and biological functions from the differentially expressed miRNAs in PBC. The results demonstrate that the unique expression pattern of serum miRNAs can serve as a noninvasive biomarker for the diagnosis of PBC.

## Results

### Global analysis of miRNAs by deep sequencing

Circulating miRNAs were detected from human serum in 10 patients diagnosed with PBC and 15 non-PBC subjects with CH-B, CH-C and healthy controls, using Illumina GA IIx sequencing. Detailed clinical information is shown in table 1. We analyzed three samples by single-end deep sequencing on one lane and obtained about 12 million 32-mer short RNA reads on average per sample. After trimming the reads to exclude adaptor and tag sequences by using cutadapt, 9,245,752 high-quality reads per sample were subjected to analysis [27]. The mapping rates to miRBase were 16.60% on average and those to hg19 were 81.66% (Table 2).

**Table 1:** Clinical data for patients enrolled in Illumina sequencing analysis.

Patient	Sex	Age	Biopsy finding^a^	T-bil (mg/dl)	ALT (IU/l)	ALP (IU/l)	Albumin (g/dl)	PT-INR	ANA	AMA	M2 (index)	HBV-DNA (Log copies/ml)	HCV-RNA (LogIU/ml)	Past treatment^b^
PBC-1	M	60	II	0.5	36	404	3.8	1.00	-	1∶80	128			URSO (−)
PBC-2	M	65	I	1.2	54	485	4.1	1.05	-	1∶160	125			URSO (−)
PBC-3	F	61	II	1.2	38	478	4.0	0.97	-	1∶20	89.5			URSO (−)
PBC-4	F	51	III	0.8	71	798	3.8	0.96	-	-	47.9			URSO (−)
PBC-5	F	62	II	0.6	26	407	4.0	0.99	-	1∶20	102			URSO (−)
PBC-6	F	61	I	0.6	38	527	4.2	0.91	-	-	111.4			URSO (−)
PBC-7	F	55	II	1.0	53	800	4.2	1.02	1∶80	-	89.9			URSO (−)
PBC-8	F	52	II	0.9	28	525	3.7	0.94	1∶80	-	-			URSO (−)
PBC-9	F	72	I	1.1	42	666	3.4	1.00	-	1∶80	143.9			URSO (−)
PBC-10	F	64	I	0.7	38	513	4.0	0.88	>2561	1∶80	79.4			URSO (−)
CH-C-1	M	57	2	0.6	22	240	4.4	0.91					5.4	IFN (+)
CH-C-2	F	55	3	1.5	73	403	4.0	1.06					5.1	IFN (+)
CH-C-3	F	61	1	1.1	12	179	4.2	1.11					5.2	IFN (+)
CH-C-4	F	49	2	0.8	42	261	3.6	1.05					6.3	IFN (−)
CH-C-5	F	53	2	0.8	27	257	4.0	0.93					6.9	IFN (+)
CH-B-1	F	35	1	0.7	585	283	4.1	1.03				9.1		NA (−)
CH-B-2	F	72	2	0.9	64	211	3.8	1.03				3.4		NA (−)
CH-B-3	F	38	2	0.8	283	268	3.1	0.99				8.8		NA (−)
CH-B-4	F	43	3	0.6	87	239	3.7	1.01				7.4		NA (−)
CH-B-5	M	47	1	0.6	40	156	4.1	1.12				5.9		NA (−)
Healthy-3	M	37		0.6	24	252	4.2	1.02						
Healthy-4	F	49		0.7	21	274	4.1	1.08						
Healthy-5	F	33		0.6	18	173	3.9	1.12						
Healthy-7	M	26		0.8	35	218	4.3	1.08						
Healthy-8	M	28		0.9	22	238	4.2	1.04						

a We used the Scheuer score in PBC and fibrosis score of histological activity index (HAI) in CH-B and CH-C [53] [54] [55].

b URSO is the abbreviation for ursodeoxycholic acid, IFN for interferon and NA for nucleos(t)ide analogue.

**Table 2:** The number of small RNAs in serum detected by Illumina sequencing.

		Cutadapt		Mapping (miRBase)		Mapping (hg19)	
Sample	Total	No. of read	% read	No. of read	% read	No. of read	% read
PBC-1	9,996,912	5,912,672	59.14	582,682	9.85	5,195,131	87.86
PBC-2	17,103,184	8,147,153	47.64	837,575	10.28	6,484,515	79.59
PBC-3	11,731,105	9,001,730	76.73	873,985	9.71	5,414,933	60.15
PBC-4	12,785,162	10,700,147	83.69	1,463,341	13.68	8,227,422	76.89
PBC-5	14,732,479	9,138,080	62.03	1,125,157	12.31	7,317,178	80.07
PBC-6	12,139,379	7,256,738	59.78	1,274,462	17.56	5,612,279	77.34
PBC-7	12,895,734	10,107,477	78.38	1,067,578	10.56	7,826,537	77.43
PBC-8	18,786,941	11,711,844	62.34	967,802	8.26	8,323,607	71.07
PBC-9	11,852,431	8,980,873	75.77	1,141,912	12.71	7,593,581	84.55
PBC-10	18,224,562	12,752,337	69.97	756,930	5.94	8,912,212	69.89
CH-C-1	10,874,814	7,820,291	71.91	2,870,316	36.70	6,924,685	88.55
CH-C-2	10,242,500	8,138,815	79.46	3,250,159	39.93	6,976,754	85.72
CH-C-3	19,183,649	12,135,107	63.26	1,878,487	15.48	9,056,579	74.63
CH-C-4	18,750,568	15,952,136	85.08	1,877,654	11.77	14,829,719	92.96
CH-C-5	12,702,304	10,306,186	81.14	1,592,422	15.45	9,269,394	89.94
CH-B-1	5,861,013	4,732,185	80.74	1,126,751	23.81	3,708,378	78.37
CH-B-2	7,164,871	5,937,732	82.87	1,213,392	20.44	5,076,436	85.49
CH-B-3	7,029,349	6,357,274	90.44	858,562	13.51	5,378,310	84.60
CH-B-4	8,077,025	6,788,987	84.05	1,581,475	23.29	6,142,836	90.48
CH-B-5	10,255,895	9,101,104	88.74	1,952,400	21.45	7,687,217	84.46
Healthy-3	11,111,254	7,843,011	70.59	1,248,499	15.92	6,778,655	86.43
Healthy-4	12,449,813	10,690,833	85.87	2,410,128	22.54	9,204,163	86.09
Healthy-5	13,597,339	8,914,365	65.56	2,214,635	24.84	6,571,819	73.72
Healthy-7	11,351,494	9,825,442	86.56	1,880,266	19.14	8,618,696	87.72
Healthy-8	14,349,933	12,891,294	89.84	2,320,100	18.00	11,618,231	90.12
Total	313,249,710	231,143,813	73.79	38,366,670	16.60	188,749,267	81.66

### miRNA expression profile in serum affected of PBC

**Figure 1:**
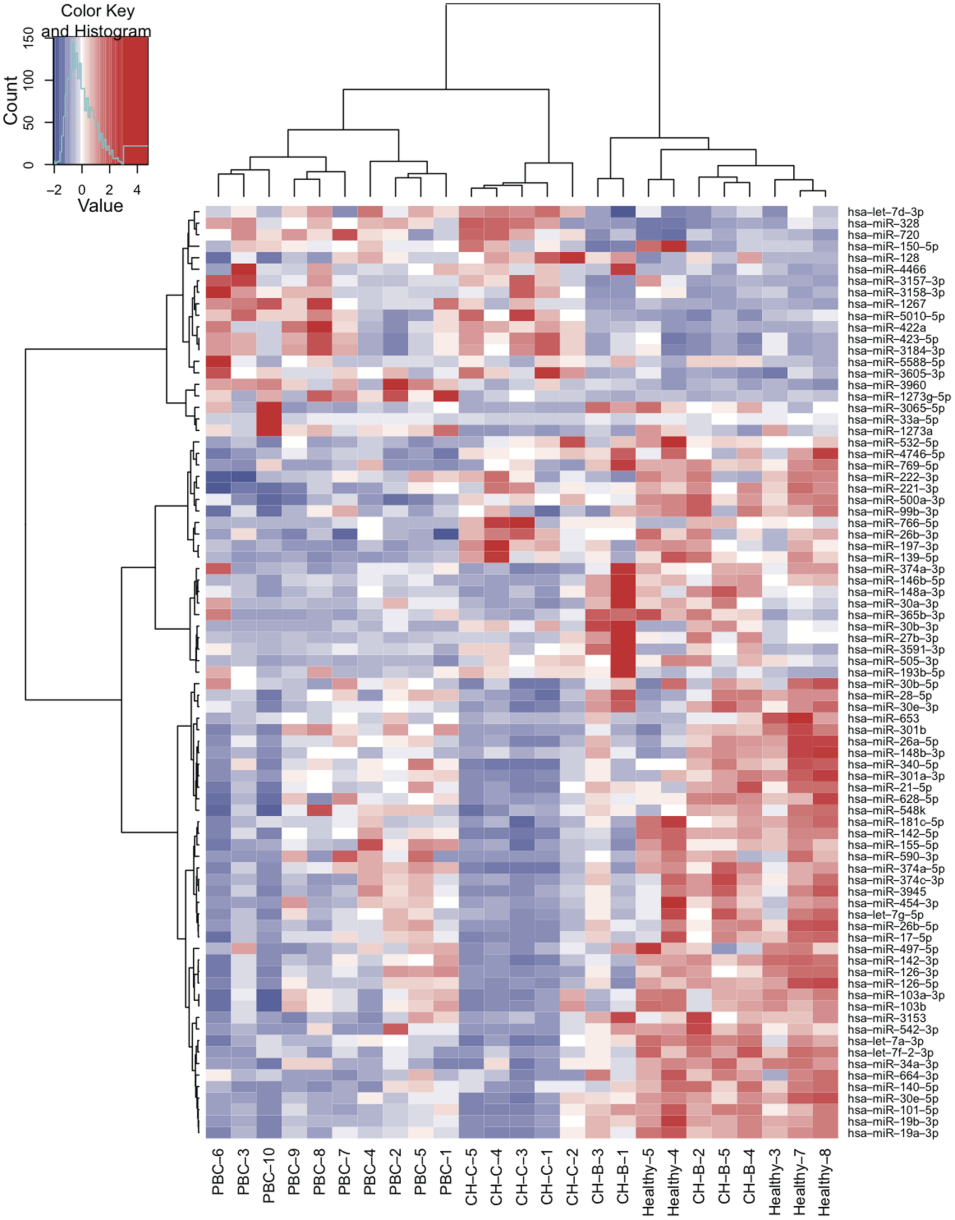
Heat map and hierarchical clustering. Individual miRNA expression were calculated by R platform and heat map was computed and described using a function of heatmap.2 in gplots. It uses hierarchical clustering with Euclidean distance; Pearson Linear Correlation and Ward's method to generate the hierarchical tree [56]. ANOVA was applied to extract differentially expressed miRNAs and adjustment of the p-value by multiple comparisons was performed by calculating FDR. Those miRNAs with FDR<0.1 were presented. The red indicates high level of miRNA expression and the blue shows low.

We normalized the differential expression of miRNA count data using the trimmed mean of M values (TMM) normalization process and the number of individual miRNA reads was standardized by the total numbers of 1,000,000 reads in each sample [28]. Comparing the 4 groups (PBC, CH-C, CH-B and healthy control), the differential expression levels of miRNA were extracted using analysis of variance (ANOVA). 1594 miRNAs were detected by deep sequencing (Table S1). Due to the small number of miRNA detections, the statistical significance of 821 miRNAs could not be determined. The ANOVA was used to determine differentially expressed miRNAs and multiple comparisons procedure was applied to compare more than one pair of means at the same time. Therefore, in statistical significance testing in the remaining 773 miRNAs, the p-value by multiple comparisons was performed by calculating False discovery rate (FDR)<0.1. The expression levels of 81 miRNAs were found to be differentially expressed in the 4 groups. Although, several types of clinical data were shown to be different from PBC, M2 negative for PBC-8, ANA positive for PBC-10 or past HBV infection for PBC-6, the histologies of all samples were characterized by PBC of chronic, nonsuppurative cholangitis affecting the interlobular and septal ducts. The heat map and hierarchical clustering demonstrated that the miRNA profiles from PBC clustered with those of CH-B, CH-C and healthy controls (Figure 1). Of note, CH-B and healthy controls were not clearly distinguished.

In the result of Illumina sequencing, 3 miRNAs were up-regulated (>2-fold) in PBC patients, with hsa-miR-1273g-5p being most enriched. The relative levels of 6 dysregulated miRNAs were down-regulated (<0.5-fold), with hsa-miR-766-5p being the smallest (Table 3).

**Table 3:** Differentially expressed miRNAs in serum from PBC patients compared with the second group (CH-C, CH-B, Healthy).

miRNA	Expression	PBC	CH-C	CH-B	Healthy	Fold change	p-value
		The mean no. of reads ±SE	The mean no. of reads ±SE	The mean no. of reads ±SE	The mean no. of reads ±SE		
hsa-miR-1273g-5p	Up	6.79±0.50	1.88±0.26	0.51±0.10	1.21±0.08	3.61	9.93E-03
hsa-miR-33a-5p	Up	6.19±1.59	0.10±0.04	1.58±0.23	2.20±0.18	2.82	4.07E-03
hsa-miR-3960	Up	11.26±0.59	4.85±0.51	3.63±0.27	1.86±0.24	2.32	4.27E-03
hsa-miR-766-5p	Down	0.17±0.04	2.92±0.53	1.55±0.10	0.64±0.12	0.27	4.61E-03
hsa-miR-505-3p	Down	5.05±0.22	16.23±0.89	26.81±3.99	16.73±1.54	0.31	3.40E-03
hsa-miR-30b-3p	Down	0.41±0.08	3.76±0.38	8.77±1.00	1.30±0.26	0.31	1.01E-03
hsa-miR-139-5p	Down	19.73±0.77	77.72±9.44	61.29±6.57	82.86±6.06	0.32	6.86E-03
hsa-miR-197-3p	Down	226.99±10.32	1067.05±106.41	589.88±60.38	823.16±66.17	0.38	7.76E-03
hsa-miR-500a-3p	Down	36.01±1.66	74.61±1.95	86.52±5.35	99.59±3.00	0.48	2.29E-03

### miRNA validation study

**Figure 2:**
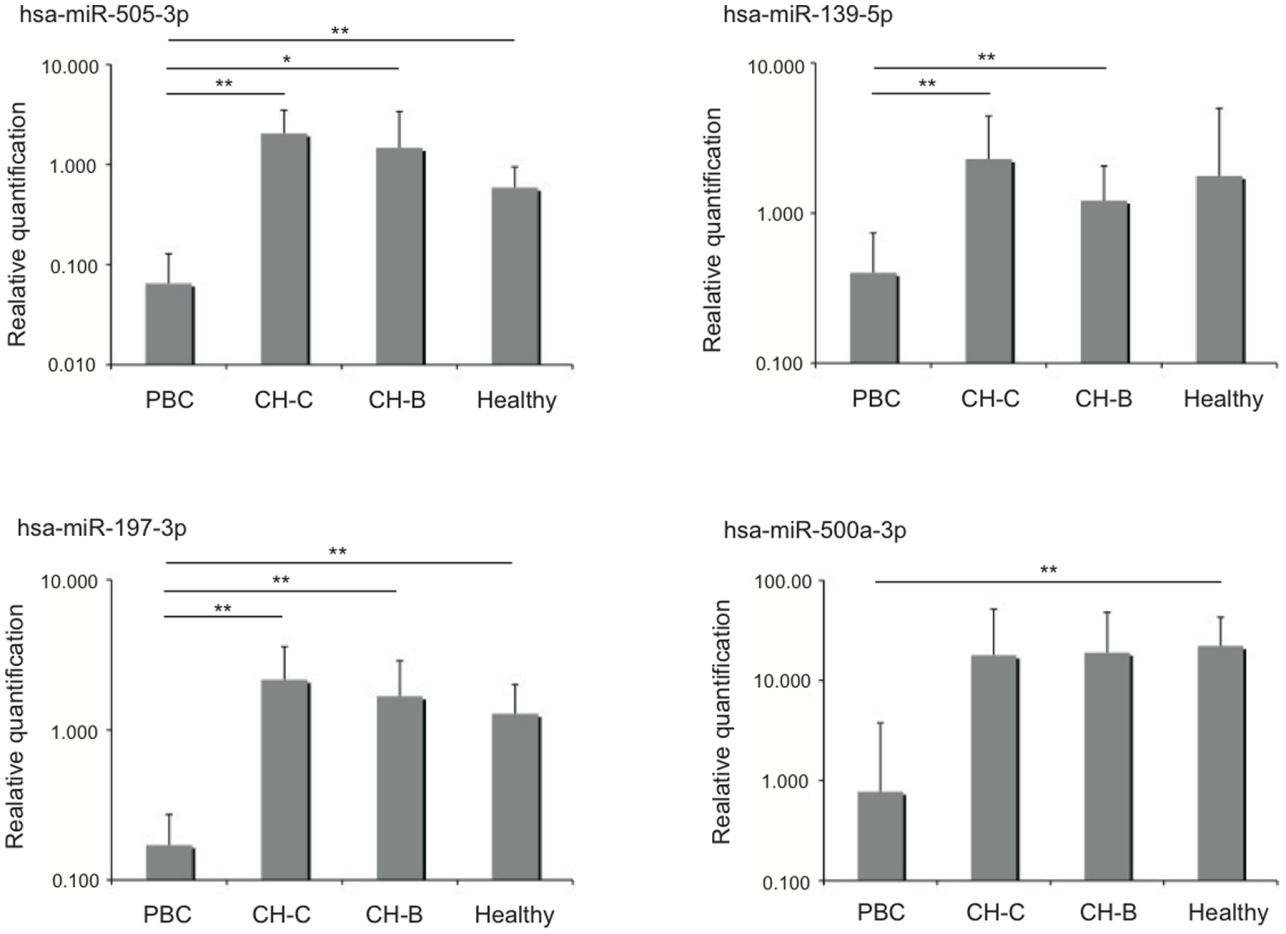
Validation of deep sequencing results for selected miRNAs. We have registered 10 samples in each group listed on Table S3. The threshold cycle for each miRNA primer/probe set were normalized with spiked in cel-miR-39 primer/probe pair and compared to CH-C-5. Result for normally distributed continuous variables are given as means and compared between groups by Student's t-test. Results for non-normally distributed continuous variables are summarized as medians and were compared by Mann-Whitney U test. Statistical significance indicates by one asterisk (p<0.05) and two (p<0.01).

We used qRT-PCR to verify the data obtained from the Illumina sequencing. The relative expression levels from 9 differentially expressed miRNAs were analyzed with the TaqMan MicroRNA assay (Applied Biosystems) or miRCURY LNA microRNA PCR system (Exiqon). In addition to the 25 samples used for Illumina deep sequencing, 10 cases of miRNA expression in PBC, CH-C, CH-B and healthy controls were quantified (Table 4). The expression levels of selected miRNAs detected by qRT-PCR were normalized to spiked-in cel-miR-39 and presented as fold-changes (2^−ΔΔCt^) above those of the CH-C-5. Among 9 miRNAs, the quantity of four miRNA expressions could be determined. The circulating levels of hsa-miR-505-3p, miR-197-3p and miR-500a-3p (p<0.01) were significantly decreased in PBC compared with healthy controls and the expression levels of hsa-miR-505-3p, miR-139-5p and miR-197-3p were significantly reduced compared with the viral hepatitis group (Figure 2). The quantities of miRNA expression by qRT-PCR were supported by the data of Illumina sequencing. Of note, for samples with only trace amounts, quantification of the reaction products after 30 cycles is uncertain. The quantity of 5 remaining miRNA expressions indicated the Ct values under 30 cycles even in the higher amount group or were undetected.

**Table 4:** Clinical information of patients enrolled in the validation study.

	PBC (n = 10)	CH-C (n = 10)	CH-B (n = 10)	Healthy (n = 10)
Male/Female	2/8	4/6	6/4	6/4
Age range	51–72	47–70	27–72	26–62
Histological findings^a^				
Scheuer score	I (4) II (5) III (1)			
HAI		1 (1) 2 (8) 3 (1)	1 (3) 2 (4) 3 (3)	
T-bil (mg/dl)^b^	0.9 (0.5–1.2)	0.9 (0.6–1.5)	0.8 (0.5–1.1)	0.8 (0.6–1.0)
ALT (IU/l)^b^	42.4 (26–71)	47.1 (12–193)	139.1 (13–585)	25.8 (18–38)
ALP (IU/l)^b^	560.3 (404–800)	238.7 (167–403)	221.7 (111–319)	230.4 (148–302)
Albumin (g/dl)^b^	3.9 (3.4–4.2)	4.0 (3.5–4.2)	3.9 (3.7–4.4)	4 (3.8–4.3)
PT-INR^b^	0.97 (0.88–1.05)	1.02 (0.91–1.11)	1.07 (0.99–1.18)	1.07 (1.02–1.13)
AMA positivity	6			
M2 positivity	9			
HBV-DNA (Log copies/ml)^b^			6.2 (3.4–9.1)	
HCV-RNA (LogIU/ml)^b^		6.4 (5.1–7.3)		

a The numbers of patients are indicated in the parentheses.

b The range of laboratory data is indicated in the parentheses.

### miRNA target genes and Gene ontology (GO) analysis

To determine possible target genes for the differentially expressed miRNAs, we searched the miRror 2.0 database for predicted miRNA targets in human. The ranking of miRror was according to the miRror Internal Score (miRIS). This score is a balance between (i) the proportions of predicting miRNA-target prediction databases (MDBs) out of all tested MDBs and (ii) the fraction of the potentially regulated genes from the entire input genes [29]. A total of 75 genes were predicted as targets for 9 differentially expressed miRNAs by Illumina sequencing of hsa-miR-1273g-5p, miR-33a-5p, miR-3960, miR-766-5p, miR-505, miR-30b, miR-139-5p, miR-197 and miR-500a in PBC serum (Table 5). Then, these 75 genes were submitted to Database for Annotation, Visualization and Integrated Discovery (DAVID) v6.7, which was used for the GO biological process categorization, Kyoto Encyclopedia of Genes and Genomes (KEGG) pathway and BIOCARTA pathway. The predicted target genes involved biological processes such as blood circulation (5.4%), circulatory system process (5.4%), positive regulation of I-kappaB kinase/NF-kappaB cascade (4.1%), regulation of phosphoprotein phosphatase activity (2.7%), cell volume homeostasis (2.7%), regulation of phosphatase activity (2.7%) and cellular amino acid derivative catabolic process (2.7%), some cellular components such as cytosol (13.5%), cell fraction (12.2%), membrane fraction (9.5%) and protein serine/threonine phosphatase complex (4.1%), and some molecular functions such as ion binding (33.8%), Cation binding (33.8%), metal ion binding (33.8%) and transition metal ion binding (25.7%) (Table S2). The functional annotation analysis of BIOCARTA showed that the genes of catenin (cadherin-associated protein), alpha 1 and similar to breast cancer anti-estrogen resistance 1 predicted target genes of the listed miRNAs, and played a role in cell-to-cell adhesion signaling pathway (Figure S1). The KEGG pathway indicated that the genes of baculoviral IAP repeat-containing 2, protein phosphatase 3 catalytic subunit beta isoform and tumor necrosis factor ligand superfamily member 10 were related to apoptosis (Figure S2).

**Table 5:** Predicted target genes of 9 differentially expressed miRNA by Illumina sequencing in PBC.

Targets	Description	miRIS*	P-value
NM_000350	ATP-binding cassette, sub-family A (ABC1), member 4	0.250	1.83E-04
NM_000434	sialidase 1 (lysosomal sialidase) (NEU1), mRNA.	0.306	6.06E-04
NM_000491	complement component 1, q subcomponent, B chain	0.250	6.64E-04
NM_000663	4-aminobutyrate aminotransferase (ABAT), nuclear gene	0.417	9.55E-03
NM_000767	cytochrome P450, family 2, subfamily B, polypeptide 6	0.361	3.72E-03
NM_000878	interleukin 2 receptor, beta (IL2RB), mRNA.	0.250	7.10E-03
NM_001001716	nuclear factor of kappa light polypeptide gene	0.306	8.66E-03
NM_001007214	calcyclin binding protein (CACYBP), transcript variant	0.306	1.90E-03
NM_001012320	zinc finger protein 302 (ZNF302), transcript variant	0.306	3.32E-03
NM_001029997	zinc finger protein 181 (ZNF181), transcript variant	0.292	7.83E-03
NM_001033557	protein phosphatase 1B (formerly 2C),	0.250	7.51E-03
NM_001033910	TNF receptor-associated factor 5 (TRAF5), transcript	0.361	5.99E-03
NM_001034	ribonucleotide reductase M2 (RRM2), mRNA.	0.361	6.78E-03
NM_001098831	MORN repeat containing 4 (MORN4), transcript variant	0.306	4.68E-03
NM_001111125	IQ motif and Sec7 domain 2 (IQSEC2), transcript	0.361	2.49E-03
NM_001128932	cytochrome P450, family 4, subfamily F, polypeptide 11	0.361	8.47E-04
NM_001135146	solute carrier family 39 (zinc transporter), member 8	0.389	1.13E-03
NM_001136216	transmembrane protein 51 (TMEM51), transcript variant	0.250	2.75E-03
NM_001142289	mahogunin, ring finger 1 (MGRN1), transcript variant	0.361	7.27E-03
NM_001142353	protein phosphatase 3 (formerly 2B), catalytic	0.250	1.72E-03
NM_001142610	unc-51-like kinase 2 (C. elegans) (ULK2), transcript	0.347	5.97E-03
NM_001143944	LEM domain containing 2 (LEMD2), transcript variant 2,	0.250	2.08E-03
NM_001146699	RNA binding motif protein 19 (RBM19), transcript	0.306	6.81E-03
NM_001159322	phospholipase A2, group IVC (cytosolic,	0.292	1.32E-03
NM_001166	baculoviral IAP repeat-containing 2 (BIRC2), mRNA.	0.250	5.10E-03
NM_001293	chloride channel, nucleotide-sensitive, 1A (CLNS1A),	0.250	1.32E-03
NM_001337	chemokine (C-X3-C motif) receptor 1 (CX3CR1), mRNA.	0.417	1.39E-03
NM_001678	ATPase, Na+/K+ transporting, beta 2 polypeptide	0.417	4.07E-04
NM_001903	catenin (cadherin-associated protein), alpha 1, 102 kDa	0.389	3.97E-03
NM_002298	lymphocyte cytosolic protein 1 (L-plastin) (LCP1),	0.417	4.64E-03
NM_003810	tumor necrosis factor (ligand) superfamily, member 10	0.250	1.95E-03
NM_004414	regulator of calcineurin 1 (RCAN1), transcript variant	0.250	5.21E-03
NM_004642	cyclin-dependent kinase 2 associated protein 1	0.306	8.18E-04
NM_005046	kallikrein-related peptidase 7 (KLK7), transcript	0.306	7.63E-03
NM_005371	methyltransferase like 1 (METTL1), transcript variant	0.250	2.91E-03
NM_005517	high-mobility group nucleosomal binding domain 2	0.417	8.69E-03
NM_005736	ARP1 actin-related protein 1 homolog A, centractin	0.361	8.46E-03
NM_006598	solute carrier family 12 (potassium/chloride	0.361	5.04E-03
NM_014012	RAS (RAD and GEM)-like GTP-binding 1 (REM1), mRNA.	0.250	7.27E-03
NM_014452	tumor necrosis factor receptor superfamily, member 21	0.250	1.53E-03
NM_014567	breast cancer anti-estrogen resistance 1 (BCAR1),	0.250	6.32E-03
NM_014718	calsyntenin 3 (CLSTN3), mRNA.	0.250	3.78E-03
NM_014784	Rho guanine nucleotide exchange factor (GEF) 11	0.306	7.74E-03
NM_015278	SAM and SH3 domain containing 1 (SASH1), mRNA.	0.417	8.14E-03
NM_015352	protein O-fucosyltransferase 1 (POFUT1), transcript	0.472	1.47E-03
NM_016033	family with sequence similarity 82, member B (FAM82B),	0.458	3.43E-03
NM_016332	selenoprotein X, 1 (SEPX1), mRNA.	0.306	4.68E-03
NM_019072	small glutamine-rich tetratricopeptide repeat	0.417	8.71E-03
NM_019860	5-hydroxytryptamine (serotonin) receptor 7 (adenylate	0.250	7.39E-04
NM_020211	RGM domain family, member A (RGMA), mRNA.	0.250	3.32E-03
NM_021131	protein phosphatase 2A activator, regulatory subunit 4	0.403	5.94E-03
NM_021939	FK506 binding protein 10, 65 kDa (FKBP10), mRNA.	0.306	1.13E-03
NM_021943	zinc finger, AN1-type domain 3 (ZFAND3), mRNA.	0.306	1.60E-03
NM_022497	mitochondrial ribosomal protein S25 (MRPS25), nuclear	0.417	8.60E-03
NM_024025	dual specificity phosphatase 26 (putative) (DUSP26),	0.403	1.66E-03
NM_024596	microcephalin 1 (MCPH1), mRNA.	0.361	1.00E-03
NM_024637	galactose-3-O-sulfotransferase 4 (GAL3ST4), mRNA.	0.361	1.73E-03
NM_024667	vacuolar protein sorting 37 homolog B (S. cerevisiae)	0.306	3.92E-03
NM_024898	DENN/MADD domain containing 1C (DENND1C), mRNA.	0.250	7.54E-03
NM_025108	chromosome 16 open reading frame 59 (C16orf59), mRNA.	0.250	3.39E-03
NM_031287	splicing factor 3b, subunit 5, 10kDa (SF3B5), mRNA.	0.306	4.48E-03
NM_032139	ankyrin repeat domain 27 (VPS9 domain) (ANKRD27),	0.306	9.38E-03
NM_032497	zinc finger protein 559 (ZNF559), mRNA.	0.347	4.45E-03
NM_080678	ubiquitin-conjugating enzyme E2F (putative) (UBE2F),	0.347	3.60E-03
NM_138396	membrane-associated ring finger (C3HC4) 9 (MARCH9),	0.306	5.18E-04
NM_138799	membrane bound O-acyltransferase domain containing 2	0.361	7.92E-03
NM_145168	short chain dehydrogenase/reductase family 42E, member	0.306	8.59E-03
NM_147202	chromosome 9 open reading frame 25 (C9orf25), mRNA.	0.417	4.67E-03
NM_173509	family with sequence similarity 163, member A	0.361	2.90E-03
NM_175839	spermine oxidase (SMOX), transcript variant 1, mRNA.	0.361	4.28E-03
NM_178468	family with sequence similarity 83, member C (FAM83C),	0.250	4.68E-03
NM_178832	MORN repeat containing 4 (MORN4), transcript variant	0.306	4.68E-03
NM_178835	zinc finger protein 827 (ZNF827), mRNA.	0.361	5.67E-03
NM_182527	calcium binding protein 7 (CABP7), mRNA.	0.417	1.95E-03
NM_198853	tripartite motif-containing 74 (TRIM74), mRNA.	0.403	1.82E-03

* miRIS :miRror Internal Score ranges from 0 top 1 by average 2 components (number of databases and input hits).

## Discussion

MiRNA changes in the liver have been reported in diseases such as HCC or chronic viral hepatitis. However, there is only limited information about their detection in blood and their correlations in PBC patients. The current study provides the first evidence that PBC is associated with altered miRNA expression. We have demonstrated that a number of miRNAs, especially hsa-miR-505-3p and miR-197-3p, were significantly differentially expressed in patients with PBC, leading to a unique miRNA expression profile in the diseased liver. Recently, many studies have examined several PBC associations with genes and there have been significant differences in the genetic risk loci reported [21] [30]. Therefore more carefully constructed studies will be needed to clarify, the pathogenesis of PBC, and the study of these differentially expressed miRNAs could serve in identifying biomarkers or lead to a better understanding of the underlying molecular mechanism that perpetuates PBC.

In our study, miRNA Illumina deep sequencing was first used to screen 10 PBC patients' sera. We were able to match the sample's sex because of particular importance for X-linked miRNA [31]. Then, qRT-PCR was used to confirm the result of deep sequencing.

Quantitative differential expression analysis identified a 81-miRNA signature distinguishing PBC, CH-C, CH-B and healthy controls. A hierarchical clustering analysis was performed utilizing the 81-miRNAs and their patterns separated the PBC from viral hepatitis and healthy controls. In addition, there were three subgroups (PBC-1,2,4,5, PBC-3,6,10 and PBC-7,8,9) in the PBC cluster. When comparing the subgroups, the PBC-3, 6,10 group showed an expression pattern that differed from those of the other two subgroups. As for the clinical background, PBC-6 patient that had been infected with hepatitis B virus were HBsAg negative and anti-HBc and anti-HBs positive, and PBC-10 patient had positive antinuclear antibodies (ANA) titers of 1∶2560 or greater, while other patients had no serological evidence of HBV infection and no positive ANA titers of 1∶160 or greater. However, there was no clear difference between the three subgroups in terms of the clinical stage. At present, there is no conclusive proof whether clinical or pathological differences can be found in clustering subgroups.

Nine miRNAs were confirmed to be significantly differentially expressed between the PBC group and viral hepatitis group or healthy control by Illumina deep sequencing. Among these 9 miRNAs, the serum levels of hsa-miR-505-3p and miR-197-3p were significantly lower in patients with PBC than in those with viral hepatitis and healthy controls, hsa-miR-139-5p was lower in patients with PBC than in those with viral hepatitis and miR-500a-3p were lower in patients with PBC than in healthy controls. Of note, we conducted qRT-PCR on the sera of some PBC who had already been treated with ursodeoxycholic acid. The serum levels of miRNA showed improvements in some samples (data not shown). Accordingly, quantifying these miRNAs may yield reliable diagnostic information. However, one problem is that the quantification of miRNA in this study used standardization by the total numbers of 1,000,000 reads in the deep sequencing or a comparative method in qRT-PCR. In other words, it was assumed that the same amount of miRNA was contained in each serum sample. Therefore, if one miRNA is quantified in a single specimen, we will not be able to accurately assess the result.

Specific circulating miRNA profiles have been reported for various diseases [32] [5] [33] [34] [35]. These circulating miRNA profiles have been described as correlating with differentially expressed miRNA in diseased tissue, such as liver injured by drugs or stomach afflicted with gastric cancer [36] [37]. Moreover, some disease-specific profiles can inform both the diagnosis and prognosis [38] [39]. Therefore, to determine if any of these diferentially expressed miRNAs could lead to better understanding of the molecular mechanism that perpetuates PBC, we examined for gene targets that may be reflected by this particular miRNA expression signature. Of the several target prediction algorithms prepared, we selected mirror 2.0. There has been evidence that a seed region of miRNA positioned within a limited range in the 3′ UTR of a target gene degrades the mRNA function [40]. We predicted 75 genes as targets for 9 differentially expressed miRNAs and conducted a functional analysis of DAVID. This analysis revealed that the genes of catenin (cadherin-associated protein), alpha 1 and similar to breast cancer anti-estrogen resistance 1 predicted target genes of the listed miRNA and played a role in cell-to-cell adhesion signaling, and the genes of baculoviral IAP repeat-containing 2, protein phosphatase 3 catalytic subunit beta isoform and tumor necrosis factor ligand superfamily member 10 were related to apoptosis. The onset of autoimmune disorders with PBC can be linked to apoptosis. A previous report described that the expression of TRAIL receptors is up-regulated by an increased bile acid level and that the serum level of soluble TRAIL is elevated, which may be involved in the development and progression of PBC [41], [42]. However, further work will be required so that these miRNAs can serve not only as biomarkers but also for the elucidation of the pathogenesis of PBC.

GO analysis provides representations of biological annotations using precisely defined terms [43]. A previous report has described a number of genes involved in the signaling, regulation of I-kappaB kinase/NF-kappaB cascade and homeostasis that are associated with PBC [44] [45], [46]. Additionally, our study indicated the biological processes, cellular component and molecular functions affected by the target genes included those associated with cell or membrane fraction, various kinds of ion binding and protein serine/threonine phosphatase complex, all of which are potentially related to PBC. Further studies will need to examine the relationship between differentially expressed miRNAs, both in serum and liver tissue, and target genes, which may provide more insights into the role of miRNAs in the pathology of PBC.

In conclusion, our results indicate that sera from patients with PBC have a unique miRNA expression profile compared to viral hepatitis and healthy controls and down-regulated expression of hsa-miR-505-3p and 197-3p may represent new clinical biomarkers in PBC. This study suggests that the amounts of miRNAs in serum have potential as diagnostic and prognostic biomarkers for PBC.

## Materials and Methods

### Patients and sample processing

We included sera of 10 patients with PBC who were treatment-naïve, sera of 5 patients with CH-B, sera of 5 patients with CH-C and sera of 5 healthy controls in this study. Initially these serum samples were enrolled to be analyzed by the Illumina miRNA deep sequencing (Illumina). The diagnosis of all cases was based on internationally established criteria [23].

### Library preparation and Illumina sequencing

A ten ml venous sample was collected from each participant. The whole blood was separated into serum and cellular fractions by centrifugation at 2,500 r.p.m. for 10 min, followed by 10 min centrifugation at 10,000 r.p.m. to completely remove cell debris. The supernatant serum was stored at −20°C until analysis. Total RNA was extracted from 800 µl of serum using Trizol LS (Invitrogen, Carlsbad, CA). The libraries were constructed from total RNA using the TruSeq Small RNA Sample Prep Kit (Illumina, San Diego, CA) following the manufacturer's protocol. Briefly, RNA 3′ and 5′ adapters were ligated to target microRNAs in two separate steps. Reverse transcription reaction was conducted to the ligation products to create single stranded cDNA. The cDNA was amplified by PCR using a common primer and a primer containing the index sequence. One µl of each library was loaded on an Agilent Bioanalyzer (Agilent, Santa Clara, CA) to check the size, purity, and concentration. Libraries were sequenced on an Illumina GA IIx (SCS 2.8 software; Illumina, SanDiego, CA), with a 32-mer single end sequence. Image analysis and base calling were performed using RTA 1.8 software.

### Sequence and statistical analysis

Raw miRNA sequence reads were conducted as a quality check and the 3′ and 5′ adapter sequences were removed by cutadapt while discarding reads shorter than 20 nucleotides [27]. The sequence reads were mapped with miRBase (Release 18) and UCSC (hg19) by use of bwa (0.5.9-r16), allowing one nucleotide base mismatch [47] [48].

Digital expression levels were normalized by taking into account the length of miRNAs and the total number of miRNA reads generated in each library using TMM normalization [28]. Read counts of each identified miRNA was normalized to the total number of miRNA reads, and then the ratio was multiplied by a constant set to 1×10^6^ in this study. ANOVA was applied to extract differentially expressed miRNAs among the four groups. Adjustment of the p-value by multiple comparisons was performed by calculating FDR [49]. Those miRNAs with FDR<0.1 were extracted as differentially expressed and used in the following analysis. Hierarchical clustering was performed using an R platform and a heat map described as using a function of heatmap.2 in gplots [50].

### qRT-PCR validation study

In addition to 25 samples analyzed by Illumina sequencing, five more serum samples of CH-B, CH-C and healthy controls (a total of 10 samples in each group) were used in qRT-PCR validation study. We followed the protocol previously reported by Mitchell *et al.* to determine the endogenous miRNA levels with spiked-in miRNA. Spiked-in miRNA was designed against *Caenorhabditis elegans* microRNA-39 (cel-miR-39) (5′- UCA CCG GGU GUA AAU CAG CUU -3′) and was synthesized by Sigma Aldrich Japan [32]. After total RNA isolation from 300 µl serum, reverse transcription was conducted using a TaqMan miRNA RT kit for identification of the cel-miR-39 expression (Applied Biosystems) with 5 fmol/µl for the internal control. qRT-PCR were conducted for detection of hsa-miR-1273g-5p, miR-505-3p and miR-139-5p in 20 µl PCR reactions using TaqMan MicroRNA assay with StepOne Plus detection system at 50°C for 2 min and 95°C for 10 min, followed by 40 cycles of 95°C for 15 s and 60°C for 1 min (Applied Biosystems). For detection of hsa-miR-33a-5p, miR-3960, miR-766-5p, miR-30b-3p, miR-197-3p and miR-500a-3p expression, we used the Exiqon system. Total RNA was reverse transcribed using the miRCURY LNA™ Universal RT miRNA PCR, Polyadenylation and cDNA synthesis kit (Exiqon). cDNA diluted 50× was assayed in 10 µl PCR reactions according to the protocol for miRCURY LNA™ Universal RT miRNA PCR with StepOne Plus detection system at 95°C for 10 min, followed by 40 cycles of 95°C for 10 s and 60°C for 1 min (Exiqon). The data were analyzed by the 2^−ΔΔCt^ method.

### Statistical methods

Expression levels of the selected miRNAs detected by qRT-PCR were normalized to cel-miR-39 and presented as the fold-change (2^−ΔΔCt^) above the control (CH-C-5): ΔΔCt = (Ct_miRNA_-Ct_cel-miR-39_)_patients_-(Ct_miRNA_-Ct_cel-miR-39_)_CH-C-5_. Results for normally distributed continuous variables are given as means (±standard errors of the mean) and compared between groups by Student's t-test. Results for non-normally distributed continuous variables are summarized as medians (interquartile ranges) and were compared by Mann-Whitney U test.

### *In silico* analysis of miRNA target gene

For computational prediction of miRNA target genes, we used an algorithm: miRror 2.0 (June 2010 release, http://www.proto.cs.huji.ac.il/mirror/) [51]. MiRror 2.0 encompasses most of the available miRNA-target prediction tools covering human miRNAs. The algorithms used are collectively called miRNA-target prediction databases (MDBs): (i) PITA (Kartez); (ii) PicTar 4 (Krek); (iii) TargetRank (Nielsen); (iv) TargetScan (Lewis); (v) microCosm (John); (vi) miRanda (Betel); (vii) DIANA-microT (Maragkakis); (viii) MirZ (Hausser); (ix) miRDB (Wang); (x) RNA 22 (Miranda); (xi) MAMI (Sethupathy); (xii) miRNAMap2 (Hsu). The number of candidate genes and the number of miRNAs are indicated for each of the major MDBs. We selected as the search mode: miR2Gene; and as the search parameters of organism: human; and of selected tissue: all. Advanced parameters were inputted, cutoff: 0.01; database hits: 2; and target counts: 3. We created a list of common target genes for miRNAs. Then, these common targets were annotated by an annotation tool at the DAVID v6.7 (January 2010 release, http://david.abcc.ncifcrf.gov/) [52]. DAVID can detect functional enrichment of a gene list based on the GO terms, KEGG pathway and BIOCARTA pathway. Differences were considered significant when the P value was less than 0.05.

### Ethics statement

This study was approved by the Ethics Committee of the Tohoku University School of Medicine (2010-404) and written informed consent was obtained from each individual.

## Supporting Information

Figure S1 **The pathway of cell-to-cell adhesion signaling.** The functional annotation analysis of BIOCARTA showed that the genes of catenin (cadherion-associated protein), alpha 1 and similar to breast cancer anti-estrogen resistance 1 played roles in this pathway. The stars indicate the related genes.
(TIFF)

Figure S2 **The pathway of apoptosis.** The functional annotation analysis of BIOCARTA showed that the genes of baculoviral IAP repeat-containing 2, protein phosphatase 3 (formerly 2B), catalytic subunit, beta isoform and tumor necrosis factor (ligand) superfamily, member 10 was related to apoptosis. The gene is indicated with the stars.
(TIFF)

Table S1 **The list of differential expression levels of miRNA in each sample.**
(XLS)

Table S2 **Biological function analysis in GO terms of predicted gene targets of differentially regulated miRNAs using DAVID.**
(DOC)
