# Supplementary figures and images for: Distinct MicroRNAs Expression Profile in Primary Biliary Cirrhosis and Evaluation of miR 505-3p and miR197-3p as Novel Biomarkers
Source: PLoS One. 2013 Jun 12;8(6):e66086. doi: 10.1371/journal.pone.0066086 (PMC3680413; doi:10.1371/journal.pone.0066086)

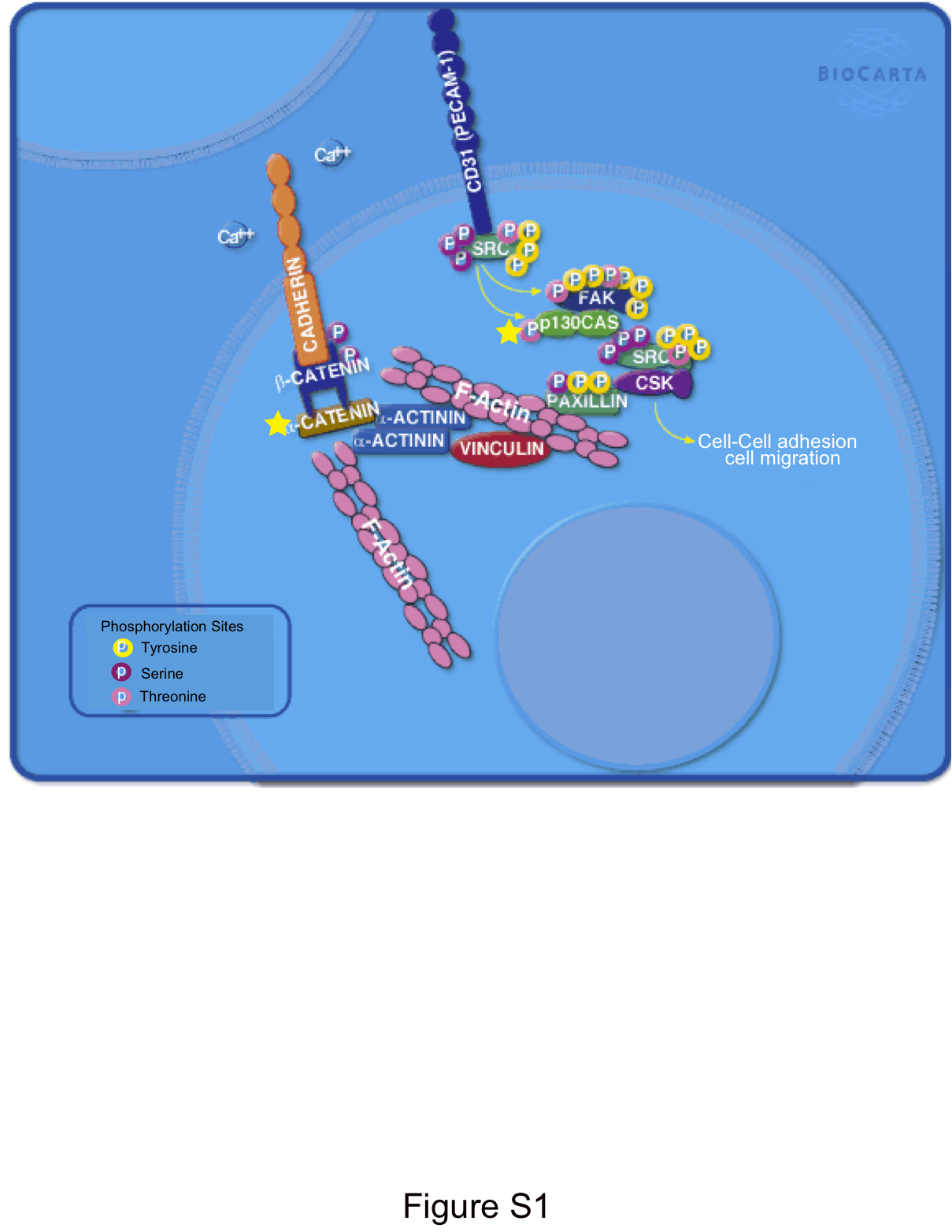

Supplement: Figure S1 — The pathway of cell-to-cell adhesion signaling. The functional annotation analysis of BIOCARTA showed that the genes of catenin (cadherion-associated protein), alpha 1 and similar to breast cancer anti-estrogen resistance 1 played roles in this pathway. The stars indicate the related genes. (TIFF) [file pone.0066086.s001.tiff]

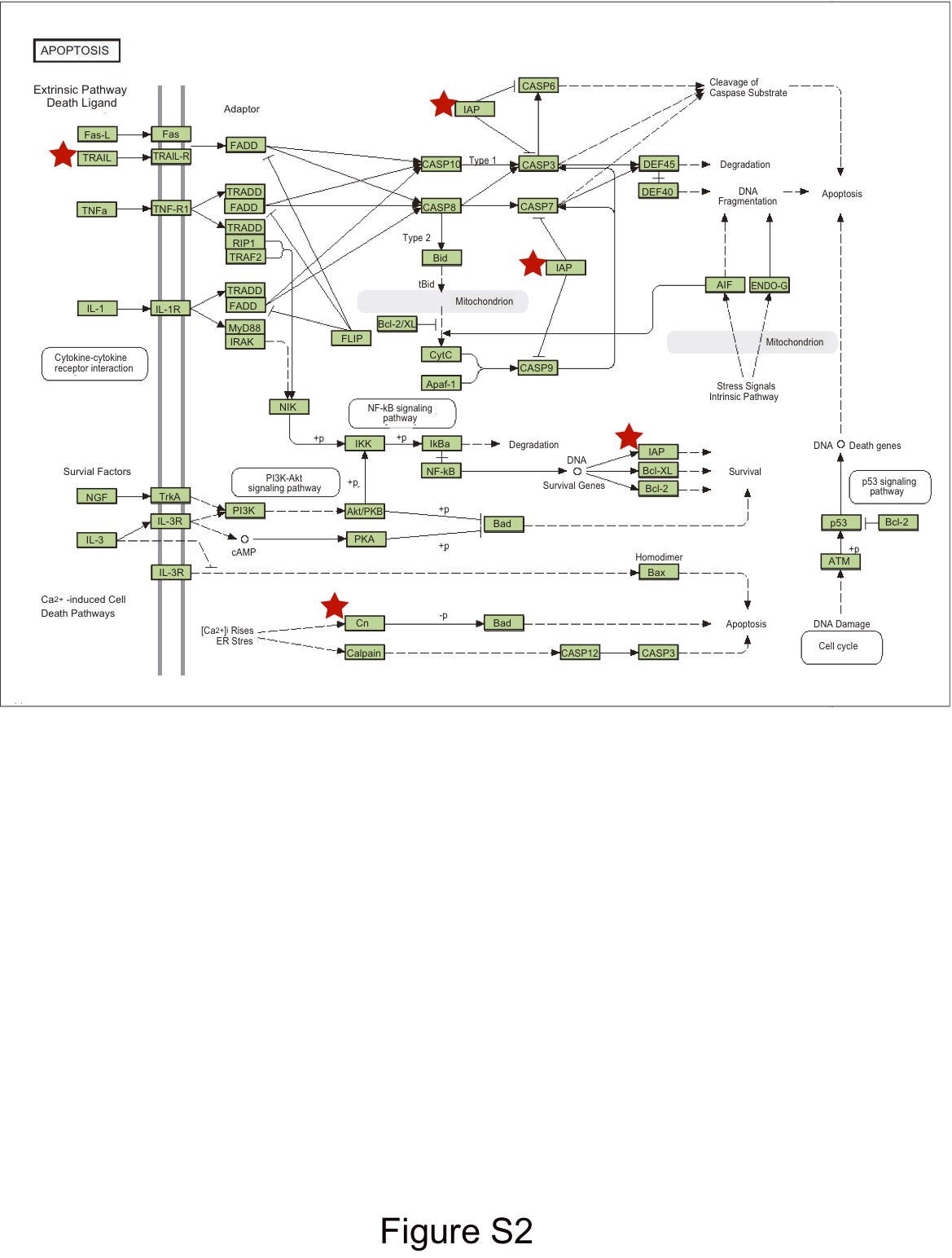

Supplement: Figure S2 — The pathway of apoptosis. The functional annotation analysis of BIOCARTA showed that the genes of baculoviral IAP repeat-containing 2, protein phosphatase 3 (formerly 2B), catalytic subunit, beta isoform and tumor necrosis factor (ligand) superfamily, member 10 was related to apoptosis. The gene is indicated with the stars. (TIFF) [file pone.0066086.s002.tiff]
