# Supplementary material for: Distinct MicroRNAs Expression Profile in Primary Biliary Cirrhosis and Evaluation of miR 505-3p and miR197-3p as Novel Biomarkers
Source: PLoS One. 2013 Jun 12;8(6):e66086. doi: 10.1371/journal.pone.0066086 (PMC3680413; doi:10.1371/journal.pone.0066086)
Supplement: Table S2 — Biological function analysis in GO terms of predicted gene targets of differentially regulated miRNAs using DAVID. (DOC) [file pone.0066086.s004.doc]

Table S2. Biological function analysis in GO terms of predicted gene targets of differentially regulated miRNAs using DAVID.

| Category | Term | Gene, n | % | P-Value |
| --- | --- | --- | --- | --- |
| Biological processes | Blood circulation | 4 | 5.4 | 4.17E-02 |
|  | Circulatory system process | 4 | 5.4 | 4.17E-02 |
|  | Positive regulation of I-kappaB kinase/NF-kappaB cascade | 3 | 4.1 | 6.10E-02 |
|  | Regulation of I-kappaB kinase/NF-kappaB cascade | 3 | 4.1 | 7.25E-02 |
|  | Regulation of phosphoprotein phosphatase activity | 2 | 2.7 | 5.25E-02 |
|  | Cell volume homeostasis | 2 | 2.7 | 5.64E-02 |
|  | Regulation of phosphatase activity | 2 | 2.7 | 7.20E-02 |
|  | Cellular amino acid derivative catabolic process | 2 | 2.7 | 7.58E-02 |
| Cellular component | Cytosol | 10 | 13.5 | 7.05E-02 |
|  | Cell fraction | 9 | 12.2 | 5.76E-02 |
|  | Membrane fraction | 7 | 9.5 | 9.42E-02 |
|  | Protein serine/threonine phosphatase complex | 3 | 4.1 | 1.06E-02 |
| Molecular functions | Ion binding | 25 | 33.8 | 5.77E-02 |
|  | Cation binding | 25 | 33.8 | 4.92E-02 |
|  | Metal ion binding | 25 | 33.8 | 4.44E-02 |
|  | Transition metal ion binding | 19 | 25.7 | 3.47E-02 |

P-value of <0.1 was considered statistically significant.
